# Postmarket Safety Actions for Novel Oncology Drugs Granted FDA’s Accelerated Approval

**DOI:** 10.1001/jamanetworkopen.2025.53764

**Published:** 2026-01-13

**Authors:** Maryam Mooghali, Joshua D. Wallach, Aaron P. Mitchell, Joseph S. Ross, Reshma Ramachandran

**Affiliations:** 1 Department of Internal Medicine, Yale School of Medicine, New Haven, Connecticut; 2 Yale Collaboration for Regulatory Rigor, Integrity, and Transparency (CRRIT), Yale School of Medicine, New Haven, Connecticut; 3 Department of Epidemiology, Rollins School of Public Health, Emory University, Atlanta, Georgia; 4 Department of Epidemiology and Biostatistics, Memorial Sloan Kettering Cancer Center, New York, New York; 5 Department of Health Policy and Management, Yale School of Public Health, New Haven, Connecticut; 6 Center for Outcomes Research and Evaluation, Yale-New Haven Health System, New Haven, Connecticut

## Abstract

This cross-sectional study examines postmarket safety actions for novel oncology drugs that received accelerated Food and Drug Administration (FDA) approval.

## Introduction

The US Food and Drug Administration (FDA) grants accelerated approval to drugs intended for serious or life-threatening conditions, allowing earlier patient access based on pivotal trials using surrogate markers as primary endpoints.^1^ More than 80% of accelerated approvals are for oncology drugs.^2^ Given uncertain efficacy at approval, FDA requires postmarket studies to confirm benefit.^1,2^ However, safety monitoring is also important to ensure that predicted benefits outweigh risks. Drugs approved through FDA’s expedited pathways, including accelerated approval, are more frequently associated with postmarketing safety actions than those approved via standard pathways,^3,4^ but more contemporaneous estimates are lacking. We examined FDA’s postmarketing safety actions for novel oncology drugs granted accelerated approval.

## Methods

In this cross-sectional study, we used the Drugs@FDA database to identify and characterize all oncology drugs granted accelerated approval from 2011 to 2020, allowing at least 4 subsequent years for FDA safety actions. Using FDA’s Project Confirm, we determined whether accelerated approval indications were converted to traditional approval or withdrawn from market. We identified FDA’s postmarketing safety actions, including warnings and precautions, boxed warnings, safety communications, and safety-related withdrawals, as of April 30, 2025 (eMethods in Supplement 1). Next, we reviewed subsequent approval packages to determine whether drugs with postmarketing safety actions later received accelerated approval for supplemental indications. Lastly, we reviewed initial accelerated approval letters to determine whether there were safety-related postmarketing requirements under FDA’s authority designated by section 505(o) of the Federal Food, Drug, and Cosmetic Act and whether safety issues were to be examined as part of accelerated approval postmarketing requirement.

Fisher exact and χ^2^ tests were performed in RStudio version 2023.09.1 + 494 (Posit), with 2-tailed *P* < .05 considered statistically significant. The Yale School of Medicine institutional review board deemed this study exempt from review because it utilized publicly available data and did not require ethics approval or patient consent, in accordance with 45 CFR S46. We followed the Strengthening the Reporting of Observational Studies in Epidemiology (STROBE) reporting guideline.

## Results

From 2011 to 2020, FDA granted accelerated approval to 52 novel oncology drugs, including 35 (67.3%) small molecules and 17 (32.7%) biologics; 22 (42.3%) were first-in-class, 45 (86.5%) designated as orphan drugs, and 36 (69.2%) designated as breakthrough therapies. Of these 52 drugs, 36 (69.2%) experienced any FDA postmarketing safety action, including 33 (63.5%) new warnings and precautions, 6 (11.5%) new boxed warnings, 4 (7.7%) drug safety communications, and 2 (3.8%) safety-related market withdrawals (Table). Median (IQR) time from approval to first safety action was 2.2 (1.3-3.8) years (Figure). Of the 36 drugs with postmarketing safety actions, 9 (25%) subsequently received accelerated approval for additional indications.

**Table.** FDA’s Postmarketing Safety Actions for Oncology Drugs Granted FDA’s Accelerated Approval, 2011-2020

FDA postmarketing safety actions	No. (%) (N = 52)				*P* value^b^
	All oncology drugs granted accelerated approval	Accelerated approval status, drugs with initial accelerated approval indication			
		Converted to traditional approval (n = 33)	Withdrawn from the market (n = 11)	Unchanged regulatory status (n = 8)^a^	
Any postmarketing safety action					
No	16 (30.8)	7 (21.2)	5 (45.5)	4 (50.0)	.14
Yes	36 (69.2)	26 (78.8)	6 (54.5)	4 (50.0)	
Time to first safety action, median (IQR) y	2.2 (1.3-3.8)	2.0 (1.1-2.6)	3.6 (2.5-4.3)	3.9 (2.9-5.4)	
Warnings and precautions					
No	19 (36.5)	7 (21.2)	8 (72.7)	4 (50.0)	.006
Yes	33 (63.5)	26 (78.8)	3 (27.3)	4 (50.0)	
No. of warnings and precautions, median (IQR)	2 (1-3)	2 (1-3)	1 (1-3)	2 (1-2)	
Boxed warning					
No new postmarketing boxed warning	46 (88.5)	29 (87.9)	9 (81.8)	8 (100.0)	.58
Existing boxed warning on approval	8 (17.4)	4 (13.8)	3 (33.3)	1 (12.5)	
No boxed warning on approval	38 (82.6)	25 (86.2)	6 (66.7)	7 (87.5)	
New postmarketing boxed warning	6 (11.5)	4 (12.1)	2 (18.2)	0	
Existing boxed warning on approval	4 (66.7)	2 (50.0)	2 (100.0)	0	
No boxed warning on approval	2 (33.3)	2 (50.0)	0	0	
Drug safety communication					
No	48 (92.3)	30 (90.9)	10 (90.9)	8 (100.0)	>.99
Yes	4 (7.7)	3 (9.1)	1 (9.1)	0	
No. of drug safety communications, median (IQR)	1 (1-1)	1 (1-2)	1 (1-1)	NA	
Safety related withdrawal					
No	50 (96.2)	32 (97.0)	10 (90.9)	8 (100.0)	.60
Yes	2 (3.8)	1 (3.0)	1 (9.1)	0	

Abbreviation: FDA, US Food and Drug Administration.

^a^ Drugs that the initial accelerated approval indication still had an accelerated approval status.

^b^ *P* values are based on χ^2^ or Fisher exact test, comparing presence or absence of each postmarketing safety actions across all three regulatory status.

**Figure.**
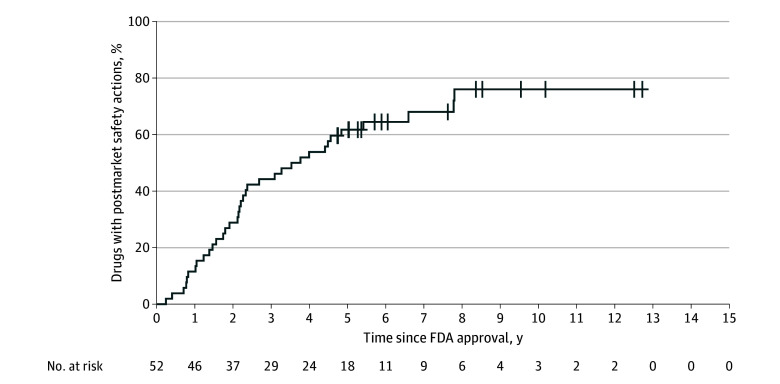
Oncology Drugs Granted FDA Accelerated Approval From 2010 to 2020 With Postmarket Safety Actions Number at risk indicates the number of drugs remaining under observation for potential safety actions at each time point, from the date of accelerated approval until the occurrence of a safety action or end of follow-up.

Drugs with accelerated approval postmarketing requirements that included safety analyses were more likely to have postmarketing safety actions than those without (92.3% [12 of 13] vs 61.5% [24 of 39]; *P* = .04), but there was no difference between those with and without safety-related postmarketing requirements (76.3% [29 of 38] vs 50.0% [7 of 14]; *P* = .09). There were no statistical differences in overall frequency of postmarketing safety action based on updated regulatory status, nor any individual safety action, except for warnings and precautions (63.5% [33 of 52] vs. 36.5% [19 of 52]; *P* = .006).

## Discussion

Among novel oncology drugs granted FDA’s accelerated approval from 2011 to 2020, two-thirds were associated with postmarketing safety actions, mostly additions of warnings and precautions, occurring at a median of 2 years after approval. Most serious safety actions were infrequent but must be interpreted amid uncertain benefit, as many oncology drugs granted accelerated approval lack confirmation of clinical benefit even after conversion to traditional approval.^5^ Drugs with safety analyses as part of their accelerated approval postmarketing requirements had higher rates of safety actions, suggesting that proactive surveillance may facilitate harm detection, particularly given the small sample size and short follow-up of pivotal studies supporting accelerated approvals.^6^ Study limitations include reliance on publicly available data and lack of a contemporaneous comparator group of oncology drugs receiving traditional approval. Although current FDA’s surveillance systems detect many safety concerns, our findings underscore the need to strengthen safety monitoring alongside efforts to confirm clinical benefit to improve accelerated approval program, particularly considering their uncertain efficacy and vulnerability of the target population.

## Notes

Supplement 1. eMethods.

Supplement 2. Data Sharing Statement
