# Supplementary material for: Postmarket Safety Actions for Novel Oncology Drugs Granted FDA’s Accelerated Approval
Source: JAMA Netw Open. 2026 Jan 13;9(1):e2553764. doi: 10.1001/jamanetworkopen.2025.53764 (PMC12801081; doi:10.1001/jamanetworkopen.2025.53764)
Supplement: Supplement 1. — eMethods. [file jamanetwopen-e2553764-s001.pdf]

Supplemental Online Content

Mooghali M, Wallach JD, Mitchell AP, Ross JS, Ramachandran R. Postmarket safety actions for novel oncology drugs granted FDA's accelerated approval. *JAMA Netw Open.* 2026;9(1):e2553764. doi:10.1001/jamanetworkopen.2025.53764

eMethods.

This supplemental material has been provided by the authors to give readers additional information about their work.

© 2025 Mooghali M et al. *JAMA Network Open*.

eMethods.

We identified warnings and precautions and boxed warnings by reviewing original and updated drug labels. We identified Drug Safety Communications and safety-related withdrawals from the FDA's Postmarket Drug Safety Information website, Index to Drug-specific Information website, and manufacturer press releases.

Box – FDA's Definition of Safety Actions and Postmarketing Requirements

Warnings and Precautions: A section of FDAs' drug label intended to identify and describe "adverse reactions and other potential safety hazards that are serious or are otherwise clinically significant" with "implications for prescribing decisions for patient management".[1]

Boxed Warning: A section of FDA's drug label that highlights for prescribers "an adverse reaction so serious in proportion to the potential benefit", "a serious adverse reaction that can be prevented or reduced in severity by appropriate use", or a drug approved "with restrictions to ensure safe use".[1]

Drug Safety Communications: Public statements issued by the FDA to provide "important information to patients and health care professionals about new safety issues with medicines" to support informed treatment decisions.[2]

§505(o) Postmarketing Requirements: Postapproval studies required by the FDA from manufacturers to "assess known serious risks", "signals of serious risk", or "identify unexpected serious risks" related to a drug.[3] These can be issued at any time, either with FDA approval or afterward, and have specific deadlines negotiated with the manufacturer to submit a protocol, complete the study, and submit the study results.

Accelerated Approval Postmarketing Requirements: Postapproval studies that may be required by the FDA from the manufacturers as a condition of receiving accelerated approval to "verify clinical benefit". These are issued upon approval with specific deadlines for the manufacturer to submit a study protocol, complete the study, and submit study results to the FDA for consideration of either converting the drug to a traditional approval, continuing to approve the drug as an accelerated approval, or withdrawing the drug indication approval from the market.[4]

© 2025 Mooghali M et al. *JAMA Network Open*.

References

1. U.S. Food and Drug Administration. Guidance for Industry: Warnings and Precautions, Contraindications, and Boxed Warning Sections of Labeling for Human Prescription Drug and Biological Products - Content and Format. Accessed November 6, 2025. https://www.fda.gov/media/71866/download
2. U.S. Food and Drug Administration. Drug Safety Communications. Accessed November 6, 2025. https://www.fda.gov/drugs/drug-safety-and-availability/drug-safety-communications
3. U.S. Food and Drug Administration. Postmarketing Requirements and Commitments: Frequently Asked Questions (FAQ). Accessed November 6, 2025. https://www.fda.gov/drugs/postmarketing-requirements-and-commitments-introduction/postmarketing-requirements-and-commitments-frequently-asked-questions-faq
4. U.S. Food and Drug Administration. Expedited Program for Serious Conditions — Accelerated Approval of Drugs and Biologics, Guidance for Industry. Accessed November 6, 2025. https://www.fda.gov/media/184120/download

© 2025 Mooghali M et al. *JAMA Network Open*.
