# Supplementary material for: Postmarket Safety Actions for Novel Oncology Drugs Granted FDA’s Accelerated Approval
Source: JAMA Netw Open. 2026 Jan 13;9(1):e2553764. doi: 10.1001/jamanetworkopen.2025.53764 (PMC12801081; doi:10.1001/jamanetworkopen.2025.53764)
Supplement: Supplement 2. — Data Sharing Statement [file jamanetwopen-e2553764-s002.pdf]

Data Sharing Statement

Mooghali. Postmarket Safety Actions for Novel Oncology Drugs Granted FDA's Accelerated Approval, 2011-2020. *JAMA Netw Open*. Published January 13, 2026. doi:10.1001/jamanetworkopen.2025.53764

Data
Data available: No

Additional Information
Explanation for why data not available: Relevant data are available on reasonable request from the corresponding author.
